# Combined effect of transcutaneous auricular vagus nerve stimulation and 0.1 Hz slow-paced breathing on working memory

**DOI:** 10.3389/fnins.2023.1133964

**Published:** 2023-03-09

**Authors:** Qian-Qian Tian, Chen Cheng, Peng-Hui Liu, Zi-Xin Yin, Meng-Kai Zhang, Ya-Peng Cui, Rui Zhao, Hui Deng, Li-Ming Lu, Chun-Zhi Tang, Neng-Gui Xu, Xue-Juan Yang, Jin-Bo Sun, Wei Qin

**Affiliations:** ^1^Intelligent Non-Invasive Neuromodulation Technology and Transformation Joint Laboratory, Xidian University, Xi’an, Shaanxi, China; ^2^Engineering Research Center of Molecular and Neuro Imaging of the Ministry of Education, School of Life Science and Technology, Xidian University, Xi’an, Shaanxi, China; ^3^School of Electronics and Information, Xi’an Polytechnic University, Xi’an, Shaanxi, China; ^4^Guangzhou Institute of Technology, Xidian University, Xi’an, Shaanxi, China; ^5^South China Research Center for Acupuncture and Moxibustion, Medical College of Acu-Moxi and Rehabilitation, Guangzhou University of Chinese Medicine, Guangzhou, China

**Keywords:** taVNS, slow-paced breath, working memory, spatial n-back, synergistic effects

## Abstract

**Background:**

Previous research has found that transcutaneous auricular vagus nerve stimulation (taVNS) can improve working memory (WM) performance. It has also been shown that 0.1 Hz slow-paced breathing (SPB, i.e., breathing at a rate of approximately 6 breaths/min) can significantly influence physical state and cognitive function *via* changes in autonomic afferent activity. In the present study, we investigated the synergistic effects of taVNS and SPB on WM performance.

**Methods:**

A total of 96 healthy people participated in this within-subjects experiment involving four conditions, namely taVNS, SPB, combined taVNS with SPB (taVNS + SPB), and sham. Each participant underwent each intervention for 30 min and WM was compared pre- and post-intervention using the spatial and digit n-back tasks in a random order four times. Permutation-based analysis of variance was used to assess the interaction between time and intervention.

**Results:**

For the spatial 3-back task, a significant interaction between time and intervention was found for the accuracy rate of matching trials (mACC, *p* = 0.03). *Post hoc* analysis suggested that both taVNS and taVNS + SPB improved WM performance, however, no significant difference was found in the SPB or sham groups.

**Conclusion:**

This study has replicated the effects of taVNS on WM performance reported in previous studies. However, the synergistic effects of combined taVNS and SPB warrant further research.

## 1. Introduction

Working memory (WM), which is a core component of higher cognitive functions, is vital for complex mental abilities, including problem solving, reasoning, and learning (Baddeley and Hitch, 1974; Chiesa et al., 2011). Changes in WM are associated with normal neurocognitive aging and various psychiatric disorders, such as schizophrenia, attention-deficit/hyperactivity disorder (ADHD), and Alzheimer’s disease (Karatekin and Asarnow, 1998; Park and Reuter-Lorenz, 2009; Grady, 2012; van Ewijk et al., 2015; Manoach and Stickgold, 2019). In prior studies, drugs (Zhao et al., 2019) and behavioral interventions (Hargreaves et al., 2015; Ackermann et al., 2018) have been used to improve WM. In recent years, non-invasive neuromodulation techniques, such as transcranial direct current stimulation (tDCS) (Andrews et al., 2011; Zivanovic et al., 2021), transcranial alternative current stimulation (tACS) (Benussi et al., 2021), transcranial random noise stimulation (tRNS) (Murphy et al., 2020), and transcranial magnetic stimulation (TMS) (Hulst et al., 2017), have gradually become mainstream clinical treatment approaches for cognitive modulation. Of the various neuromodulation methods available, our group has mainly focused on the effects of transcutaneous auricular vagus nerve stimulation (taVNS), a peripheral nerve stimulation technique, on cognition—especially WM performance. This method offers three main benefits. Firstly, taVNS is cost effective, low risk, convenient and acceptable for most people (Badran et al., 2019). Secondly, a potential mechanism explaining the influence of taVNS on cognitive functions has been proposed, since the vagus nerves are intimately linked to perception and regulation of the central nervous system (CNS) with “bottom-up” functions in cognition and clinical disorders (Aniwattanapong et al., 2022). Some brain imagining studies further have shown that taVNS influences a range of cortical and subcortical regions important for cognitive functions, including the contralateral post-central gyrus, bilateral insula, frontal cortex, right operculum, left cerebellum, insula, hippocampus, amygdala, and thalamus (Yakunina et al., 2017; Badran et al., 2018). Thirdly, several studies have reported that taVNS improves several cognitive abilities (Beste et al., 2016; Colzato et al., 2018; Fischer et al., 2018; Adair et al., 2020; Neuser et al., 2020). In the last 2 years, our team has confirmed the positive effects of taVNS on WM performance in healthy cohorts (Sun et al., 2021a; Zhao et al., 2022).

Recent innovations in medicine and wellness are rediscovering and validating various practices such as yoga, meditation, and breathing techniques from traditional cultures that may confer physiological and psychological benefits (Carter and Carter, 2016; Niu et al., 2019). Among these, breath-based practices, especially slow-paced breath (SPB)-based techniques, a major component of Eastern practices, have attracted attention. These practices have gradually become “Westernized” and are now routinely suggested for better health and emotion, and cognitive functioning (Brown and Gerbarg, 2005). For example, breath qigong, a traditional Chinese exercise, is considered a form of mind-body medicine that coordinates gentle exercise with relaxation through slow breathing and meditation. Recent finding support that breath qigong improves cognitive performance in patients with vascular cognitive impairment (Niu et al., 2019). Moreover, slow and rhythmic breath-based meditation (e.g., Sudarshan Kriya) has shown significant effects on reducing post-traumatic stress disorder (PTSD) symptoms, hyperarousal, and anxiety in United States military combat veterans (Seppala et al., 2014; Schulz-Heik et al., 2022), and breath-based mindfulness has been shown to have a positive influence on participants’ cognitive performance—especially WM performance (Mrazek et al., 2012; Quach et al., 2016). Even a simple slow breathing intervention can improve participants’ heart rate variability (HRV) and multitasking test performance (Bonomini et al., 2020). Taken together, this evidence suggests that slow rhythmic breathing has the potential to restore or improve cognitive function. However, some studies have reported that the influence of SPB depends on long-term training and that a single intervention did not a induce significant effect (Goldberg et al., 2021; Quek et al., 2021). Thus, it is important to explore the immediate effect of SPB intervention and further improve the effects of SPB.

Investigations into the physiological benefits of SPB have uncovered significant effects on the respiratory, cardiovascular, cardiorespiratory, and autonomic nervous systems (Russo et al., 2017). Numerous studies suggest that a slow breathing rate of around 6 breaths/min leads to cardiorespiratory coupling, which can induce greater arterial oxygen saturation, hemodynamic fluctuations, and respiratory sinus arrhythmia (RSA) sensitivity, compared to breathing at a typical rate (Bernardi et al., 1998; Chang et al., 2013; Ben-Tal et al., 2014; Li et al., 2018). These changes may have a broad influence on both physical state and mental functions. Furthermore, both arms of the autonomic nervous system, i.e., the parasympathetic and sympathetic nervous systems, are under control of the central respiratory centers. Specifically, autonomic outflows are inhibited during inspiration and disinhibited during expiration, which is termed respiratory gate theory (Badra et al., 2001; Eckberg, 2003). Prior studies have found that the stimulation effects of expiration on sympathetic bursts are significantly smaller than those on parasympathetic action, and that the greatest parasympathetic activity occurs at a low respiratory frequency (about 6 breaths/min) (Eckberg, 2003; Elghozi and Julien, 2007; Wang et al., 2013); in the other words, vagal nerves are the primary parasympathetic efferent activated by SPB. Expiration is thus important for maintaining a healthy physical and mental state in humans and may have a similar influence as taVNS. Moreover, since changes in autonomic afferent activity are believed to be the primary mechanism of SPB’s effects on psychophysiological state (Noble and Hochman, 2019), SPB and taVNS may interact in the CNS, where signals from both autonomic afferents and the auricular branch of the vagus nerves are processed and integrated (Baekey et al., 2010), leading to larger modulative effects on cognitive performance—especially WM performance. Considering the unique effects of taVNS and SPB on cognition and their potential interaction, employing them simultaneously may lead to greater improvement in WM performance, i.e., a synergistic effect. Of note, a recent review commented that combining taVNS with SPB might produce positive effects *via* both interventions, which is certainly worth exploring (Szulczewski, 2022).

To the best of our knowledge, no prior studies have explored the role of combined taVNS and SPB on cognitive functions, specifically WM. Thus, the current study used four intervention conditions, i.e., taVNS, SPB, taVNS + SPB, and a sham condition, to ascertain whether taVNS and SPB modulate WM performance in healthy adults and lead to a syncretic effect.

## 2. Materials and methods

### 2.1. Participants

A total of 96 healthy students (49 females, average age = 21.07 ± 1.96 years, range 18–25 years) from Xidian University and Xinxiang Medical University took part in this experiment. To be eligible, participants were required to be right-handed and have normal or corrected-to-normal vision. The exclusion criteria included a self-reported history of a diagnosed WM deficit (e.g., Attention Deficit Hyperactivity Disorder, ADHD), neurological diseases (e.g., epilepsy), respiratory problems (e.g., cold or bronchitis), head injury, regular use of a drug or medication that could influence heart rate (e.g., anti-anxiety drugs), and smoking. No participants reported ear injuries, drinking, smoking, or taking drugs 24 h before the experiment. Before each intervention, participants completed the state subtest of State-Trait Anxiety Inventory (STAI-S); participants with a score >54 were excluded due to high anxiety levels (Kvaal et al., 2005). Two participants did not complete the full experiment and one student did not reach 0.1 Hz (6 breaths/min) more than 85% of the time and was excluded. Thus, 93 participants completed full experiment and were included in data analysis. Participants received detailed information about the experiment and provided written consent, in line with the Declaration of Helsinki. This study was approved by the institutional research ethics committee of Xijing Hospital of the Air Force Medical University.

### 2.2. Experimental design

**FIGURE 1:**
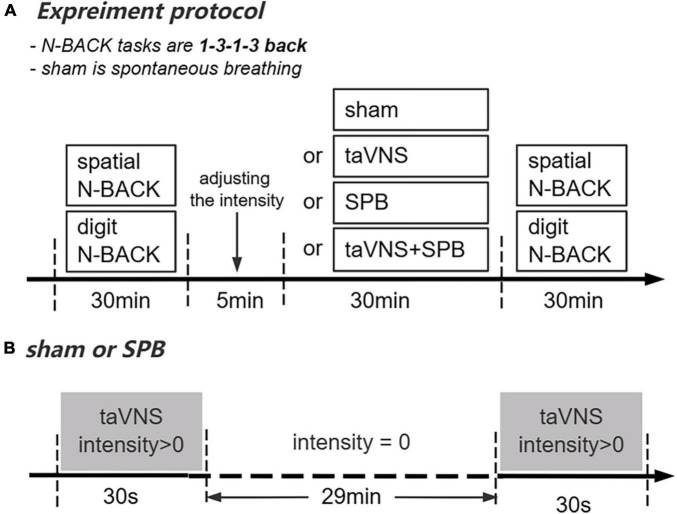
Overview of the present study. **(A)** Experimental protocol. **(B)** Current stimulation delivered in the SPB and sham conditions.

As part of the within-subject design, participants completed four independent experimental sessions separated by at least 4 days (7 ± 3 days). Before the first session, participants came to the laboratory to familiarize themselves with the protocol, behavioral tasks (spatial and digit n-back tasks), and SPB training. In this session, participants had to complete the WM tasks until they reached an accuracy rate >60%. Next, they completed 10 min 0.1 Hz SPB (i.e., 6 breaths/min) training. To determine participants’ respiratory frequency, electrocardiograph (ECG) data were recorded using ECG equipment (XD-Kerfun ECG-N02, Xi’an Kerfun Medical Co., Ltd) with a ADS1292R chip, which can collect both ECG and respiration signal. In this session, participants needed to maintain a respiratory frequency rate between 0.09 and 0.11 Hz in >90% of the training time (i.e., 9 min); otherwise, they completed training again. In the formal sessions, participants completed the cognitive tests. The current intensity threshold was tested during all four sessions to achieve a “moderately strong, but not painful sensation” corresponding to a target score of 4–5 on a 0–10 scale (Sclocco et al., 2020; Sun et al., 2021a; Sevoz-Couche and Laborde, 2022). The four 30 min experimental interventions (sham, SPB, taVNS, taVNS + SPB) were delivered in a balanced order; thus, there were 24 intervention orders, each of which was performed by four participants. Participants’ ECG data were recorded during this process. In the taVNS group, participants received taVNS current and breathed at a normal pace while watching neutral pictures on the computer. In the sham group, participants received only 30 s of current at the beginning and end of the test and breathed at a normal pace while watching neutral pictures on the computer. In the SPB and taVNS + SPB groups, participants were instructed to breathe along with a standardized visual breathing cursor on the computer, which was set at a rate of 6 breaths/min. Participants wore a breathing band to monitor their respiratory frequency. In the SPB group, participants wore taVNS stimulation equipment but only received 30 s of current at the beginning and end of the intervention. In the taVNS + SPB group, participants simultaneously received the taVNS and SPB interventions. After completing all trials, participants performed the cognitive tasks again (see Figure 1A).

### 2.3. Cognitive tests

Participants completed two WM tasks: spatial and digit n-back tasks. The tasks paradigm was the same as in our previous study (Sun et al., 2021a). Briefly, each task comprised four blocks (1-back, 3-back, 1-back, 3-back) with 72 experimental trials per block. Each block was separated by a 30 s rest period. For the spatial 1, 3-back task, participants were instructed to press “F” when the presented symbol (“*”) was the same as that shown one or three trials earlier, and to otherwise press “J.” For the digit 1, 3-back task, the stimuli were changed from a symbol “*” to nine Arabic numbers (1–9). Participants were required to judge whether the number was the same as that presented one or three trials earlier. One third of trials were matching and the maximum response time was 1600 ms.

### 2.4. taVNS stimulation equipment and parameters

The electrical stimulation equipment (XD-Kerfun BS-VNS-001) used in this study was an upgraded version of that successfully used in our previous research (Sun et al., 2021b; Shen et al., 2022). The anode and cathode of taVNS were both placed on the left cymba conche with the cathode inside and 0.5 cm apart from the anode. The electrical stimulation waveform was a single-phase rectangular pulse with a pulse width of 500 ms and frequency of 25 Hz. For the taVNS and taVNS + SPB interventions, the current was delivered with a cycle of 30 s on/30 s off in 30 min. This cycle of stimulation was delivered for the first and last 30 s on in sham and SPB group to blind participants about the intervention (see Figure 1B).

### 2.5. Statistical analysis

The primary WM outcome measures were accuracy in matching trials (mACC) and mismatching trials (mismACC), and reaction time in accurate trials (aRT) on the spatial and digit n-back tasks. Permutation-test-based one-way analysis of variance (ANOVA) and two-way ANOVA with 100,000 random samples were employed to test the main effects of stimulation and the interaction between time and stimulation. Firstly, one-way ANOVA was used to confirm that the stimulation conditions did not differ significantly in accuracy or response time at baseline (both *p* > 0.05). Next, the effects of stimulation on accuracy and reaction time were assessed separately using 2 × 4 repeated measures ANOVA with stimulation (taVNS, SPB, taVNS + SPB, and sham) and time (baseline and post-test) as within-subject factors. Subsequently, for each significant interaction effect, *post hoc* analysis using the paired *t*-test was employed for each intervention condition to examine changes in WM performance over time (baseline, post-test). All analyses were performed using R 4.1.3 software. In cases of multiple comparisons, Bonferroni’s correction was applied.

## 3. Results

### 3.1. Subjective sensation and baseline performance

At the end of each session, participants used the Numeric Rating Scale (NRS) to quantify their level of pain sensation during stimulation. There was no significant difference in subjective sensation evoked by the four stimulation conditions (*p* > 0.05). There also was no significant difference between the four stimulation conditions at baseline for the spatial and digit n-back tasks (*p* > 0.05).

### 3.2. Spatial 1-back working memory performance

The main effects of stimulation were not significant for aRT [*F*_(3, 276)_ = 1.10, *p* = 1.00], mACC [*F*_(3, 276)_ = 0.30, *p* = 1.00], or mismACC [*F*_(3, 276)_ = 0.28, *p* = 1.00]. The main effects of time were significant for aRT [*F*_(1, 92)_ = 53.73, *p* < 0.001] and mACC [*F*_(1, 92)_ = 26.95, *p* < 0.001], but not mismACC [*F*_(1, 92)_ = 1.84, *p* = 1.00]. These results indicate that participants had a faster reaction time in the post-test of the spatial 1-back task but that the accuracy rate in matching trials decreased. The interaction between time and stimulation was not significant for aRT [*F*_(3, 276)_ = 0.27, *p* = 1.00], mACC [*F*_(3, 276)_ = 1.74, *p* = 1.00], or mismACC [*F*_(3, 276)_ = 2.41, *p* = 0.63] (see Table 1).

**TABLE 1:** Means, standard deviations, and two-way ANOVAs results of spatial n-back tasks.

		Spatial 1-back			Spatial 3-back		
**Descriptive statistics**		**aRT**	**mACC**	**mismACC**	**aRT**	**mACC**	**mismACC**
**Stimulation**	**Time**	**Mean ± SD**	**Mean ± SD**	**Mean ± SD**	**Mean ± SD**	**Mean ± SD**	**Mean ± SD**
taVNS	Baseline	470.1 ± 67.7	0.913 ± 0.065	0.978 ± 0.020	552.2 ± 122.6	0.831 ± 0.112	0.958 ± 0.050 0.958 ± 0.05 0.958 ± 0.05
	Post-test	455.9 ± 77.8	0.898 ± 0.080	0.983 ± 0.018	540.5 ± 128.6	0.856 ± 0.113	0.961 ± 0.045
SPB	Baseline	462.2 ± 70.3	0.915 ± 0.064	0.982 ± 0.016	552.8 ± 125.3	0.837 ± 0.127	0.961 ± 0.051
	Post-test	448 ± 61.7	0.888 ± 0.097	0.977 ± 0.055	533.4 ± 114.4	0.831 ± 0.136	0.958 ± 0.062
taVNS + SPB	Baseline	463.1 ± 71.7 446.7 ± 65.5	0.912 ± 0.061	0.981 ± 0.018	552.3 ± 123.1	0.832 ± 0.114	0.964 ± 0.032
	Post-test	446.7 ± 65.5	0.888 ± 0.082	0.983 ± 0.016	533.2 ± 116.0	0.862 ± 0.097	0.968 ± 0.035
Sham	Baseline	465.7 ± 73.9 447.1 ± 65.6	0.908 ± 0.070	0.974 ± 0.055	548.1 ± 121.7	0.839 ± 0.119	0.962 ± 0.044
	Post-test	447.1 ± 65.6	0.901 ± 0.078	0.984 ± 0.015	517.6 ± 113.0	0.834 ± 0.117	0.967 ± 0.039
**Permutation-based RM ANOVA test**							
Time		*F*_(1, 92)_ = 53.73***	*F*_(1, 92)_ = 26.95***	*F*_(1, 92)_ = 1.84	*F*_(1, 92)_ = 29.96***	*F*_(1, 92)_ = 7.42	*F*_(1, 92)_ = 1.36
Stimulation		*F*_(3, 276)_ = 1.10	*F*_(3, 276)_ = 0.30	*F*_(3, 276)_ = 0.28	*F*_(3, 276)_ = 0.76	*F*_(3, 276)_ = 0.54	*F*_(3, 276)_ = 1.12
Time × Stimulation		*F*_(3, 276)_ = 0.27	*F*_(3, 276)_ = 1.74	*F*_(3, 276)_ = 2.41	*F*_(3, 276)_ = 1.53	*F*_(3, 276)_ = 4.89*	*F*_(3, 276)_ = 0.56

One asterisk indicates a corrected *p*-value smaller than 0.05. Three asterisks indicate a corrected *p*-value smaller than 0.001. All *p*-values were corrected by Bonferroni’s correction. SD means standard deviation; RM ANOVA means repeated measures analysis of variance; bold numbers represent significant results.

### 3.3. Spatial 3-back working memory performance

**FIGURE 2:**
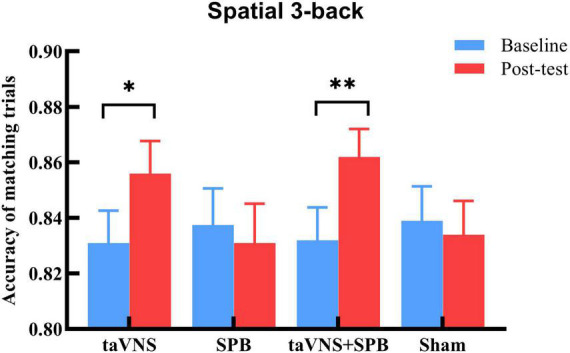
Accuracy rate of matching trials (mACC) in spatial 3-back trials. *p-value < 0.05, **p-value < 0.01.

The main effects of stimulation were not significant for aRT [*F*_(3, 276)_ = 0.76, *p* = 1.00], mACC [*F*_(3, 276)_ = 0.54, *p* = 1.00], or mismACC [*F*_(3, 276)_ = 1.12, *p* = 1.00]. The main effects of time were significant for aRT [*F*_(1, 92)_ = 29.96, *p* < 0.001], but not for mACC [*F*_(1, 92)_ = 7.42, *p* = 0.09], or mismACC [*F*_(1, 92)_ = 1.36, *p* = 1.00], which suggests that participants had a faster reaction time in the post-test of the spatial 3-back task. The interaction between time and stimulation was not significant for aRT [*F*_(3, 276)_ = 1.53, *p* = 1.00] or mismACC [*F*_(3, 276)_ = 0.56, *p* = 1.00], but was significant for mACC [*F*_(3, 276)_ = 4.89, *p* = 0.03] (see Table 1). *Post hoc* analysis suggested that, compared with baseline, there was a significant increase in the taVNS [*t*(92) = 3.000, *p* = 0.021] and taVNS + SPB [*t*(92) = 3.599, *p* = 0.002] conditions, but not the SPB [*t*(92) = 0.687, *p* = 1.000] and sham [*t*(92) = 0.621, *p* = 1.00] conditions (see Table 2 and Figure 2). These results suggest that both taVNS and taVNS + SPB stimulation improved WM accuracy.

**TABLE 2:** *Post hoc* of significant time × stimulation interactions of spatial 3-back task.

Task	Comparison	Stimulation	Spatial 3-back task			
			**Estimate**	**SD**	***t*-test**	*p*-value*
mACC	Post-test vs. Baseline	taVNS	0.025	0.081	*t*(92) = 3.000	**0.021**
		SPB	−0.006	0.085	*t*(92) = 0.687	1.000
		taVNS + SPB	0.030	0.079	*t*(92) = 3.599	**0.002**
		Sham	−0.005	0.083	*t*(92) = 0.621	1.000

*All *p*-values were corrected by Bonferroni’s correction. mACC is the accuracy rate in matching trials. The bolded font represents significant values.

### 3.4. Digit 1-back working memory performance

The main effects of stimulation were not significant for aRT [*F*_(3, 276)_ = 1.17, *p* = 1.00], mACC [*F*_(3, 276)_ = 0.54, *p* = 1.00], or mismACC [*F*_(3, 276)_ = 0.59, *p* = 1.00]. However, the main effect of time was significant for aRT [*F*_(1, 92)_ = 35.07, *p* < 0.001], but not mACC [*F*_(1, 92)_ = 3.62, *p* = 0.69], or and mismACC [*F*_(1, 92)_ = 0.71, *p* = 1.00]. These results indicate that participants performed faster in the post-test of the digit 1-back task. Further, the interaction between time and stimulation was not significant for aRT [*F*_(3, 276)_ = 2.96, *p* = 0.43], mACC [*F*_(3, 276)_ = 0.85, *p* = 1.00], or mismACC [*F*_(3, 276)_ = 1.06, *p* = 1.00] (see Table 3).

**TABLE 3:** Means, standard deviations, and two-way ANOVAs results of digit n-back tasks.

		Spatial 1-back			Spatial 3-back		
**Descriptive statistics**		**aRT**	**mACC**	**mismACC**	**aRT**	**mACC**	**mismACC**
**Stimulation**	**Time**	**Mean ± SD**	**Mean ± SD**	**Mean ± SD**	**Mean ± SD**	**Mean ± SD**	**Mean ± SD**
taVNS	Baseline	450.8 ± 69.7	0.908 ± 0.081	0.982 ± 0.024	522.5 ± 126.7	0.872 ± 0.110	0.965 ± 0.067 0.958 ± 0.05 0.958 ± 0.05
	Post-test	431.4 ± 59.9	0.903 ± 0.087	0.985 ± 0.017	496.2 ± 109.4	0.881 ± 0.092	0.974 ± 0.033
SPB	Baseline	440.4 ± 59.2	0.911 ± 0.068	0.981 ± 0.033	500.4 ± 104.3	0.872 ± 0.112	0.972 ± 0.047
	Post-test	432.4 ± 55.8	0.894 ± 0.076	0.981 ± 0.022	481.4 ± 89.9	0.874 ± 0.105	0.975 ± 0.037
taVNS + SPB	Baseline	443.7 ± 68.3	0.897 ± 0.093	0.982 ± 0.020	496.3 ± 95.5	0.877 ± 0.113	0.981 ± 0.033
	Post-test	434.6 ± 64.5	0.898 ± 0.075	0.985 ± 0.016	481.1 ± 81.1	0.881 ± 0.103	0.978 ± 0.027
Sham	Baseline	437.4 ± 62.4	0.907 ± 0.075	0.984 ± 0.017	492.4 ± 97.1	0.876 ± 0.102	0.972 ± 0.052
	Post-test	428.8 ± 51.8	0.900 ± 0.074	0.982 ± 0.022	478.2 ± 97.9	0.874 ± 0.097	0.978 ± 0.028
**Permutation-based RM ANOVA test**							
Time		*F*_(1, 92)_ = 35.07**	*F*_(1, 92)_ = **3.62**	*F*_(1, 92)_ = 0.71	*F*_(1, 92)_ = 41.28**	*F*_(1, 92)_ = 0.56	*F*_(1, 92)_ = 2.30
Stimulation		*F*_(3, 276)_ = 1.17	*F*_(3, 276)_ = 0.54	*F*_(3, 276)_ = 0.59	*F*_(3, 276)_ = 3.66	*F*_(3, 276)_ = 0.22	*F*_(3, 276)_ = 2.33
Time × Stimulation		*F*_(3, 276)_ = 2.96	*F*_(3, 276)_ = 0.85	*F*_(3, 276)_ = 1.06	*F*_(3, 276)_ = 1.27	*F*_(3, 276)_ = 0.28	*F*_(3, 276)_ = 1.10

Two asterisks indicate a corrected *p*-value smaller than 0.01. All *p*-values were corrected by Bonferroni’s correction. SD means standard deviation; RM ANOVA means repeated measures analysis of variance; bold numbers represent significant results.

### 3.5. Digit 3-back working memory performance

The main effects of stimulation were not significant for aRT [*F*_(3, 276)_ = 3.66, *p* = 0.13], mACC [*F*_(3, 276)_ = 0.22, *p* = 1.00], or mismACC [*F*_(3, 276)_ = 2.33, *p* = 0.82]. The main effect of time was significant for aRT [*F*_(1, 92)_ = 41.28, *p* < 0.001], but not mACC [*F*_(1, 92)_ = 0.56, *p* = 1.00], or mismACC [*F*_(1, 92)_ = 2.30, *p* = 1.00]. These results indicate that participants performed faster in the post-test of the digit 3-back task. Lastly, the interaction between time and stimulation was not significant for aRT [*F*_(3, 276)_ = 1.27, *p* = 1.00], mACC [*F*_(3, 276)_ = 0.28, *p* = 1.00], or mismACC [*F*_(3, 276)_ = 1.10, *p* = 1.00] (see Table 3).

## 4. Discussion

Previous studies have revealed that both taVNS and SPB have positive modulative effects on participants’ cognitive performance, especially WM capacity (Carter and Carter, 2016; Niu et al., 2019; Sun et al., 2021a; Zhao et al., 2022). These two interventions may exert their influence on participants’ physical health and mental state *via* autonomic nerves, especially the parasympathetic nervous system, i.e., vagal nerves. Thus, it is worth investigating the synergistic effects of these two interventions on WM. To do so, this study employed a within-subject design with four conditions (i.e., taVNS, SPB, taVNS + SPB, and sham) in a large sample. The results confirmed the ability of taVNS to facilitate spatial 3-back accuracy, as reported in our previous studies (Sun et al., 2021a; Zhao et al., 2022). However, SPB did not improve participants’ WM performance and there was no significant difference between the taVNS and taVNS + SPB interventions. To the best of our knowledge, this is the first study to directly compare the modulation efficiency of taVNS, taVNS + SPB and SPB interventions on WM performance. In the following sections, we discuss the potential reasons underlying the lack of effect of SPB and the limited synergistic effects of taVNS and SPB. We also propose some suggestions for practice and future research into the combined taVNS and SPB neuromodulation technique.

Recent studies have suggested that breathing at a rate of approximately 10 s/breath can significantly improve respiratory, cardiovascular, cardiorespiratory and autonomic nervous system functions (Russo et al., 2017), thereby regulating emotions and cognition (Carter and Carter, 2016; Mather and Thayer, 2018). However, more than one prior study has reported that a single SPB intervention did not influence WM performance (Goldberg et al., 2021; Quek et al., 2021). However, participants who maintained breath training for at least 3 months showed significant improvements in WM performance (Bonomini et al., 2020; Quek et al., 2021). Physiological studies provide further evidence that SPB can regulate the autonomic nervous system, especially parasympathetic nerves, i.e., vagal nerves. To achieve a long-term shift toward parasympathetic dominance, prolonged SPB practice is necessary, as observed in a previous study that required healthy humans to practice SPB regularly for 3 months (Pal et al., 2004). These results suggest that the effects of SPB on WM accumulate over time. Likely for this reason, long-term training based on intentionally decreasing the respiratory rate has existed throughout history in Chinese (Roth, 1997), Japanese (Komori, 2018), Indian (Saoji et al., 2019), and European (Bernardi et al., 2001) cultures. Today, many cognitive-behavioral therapies (CBT) include a slow breathing component, such as HRV biofeedback and mindfulness training, that lasts for about 3 months (Lehrer et al., 2020). However, the current study did not find any significant improvements in the SPB intervention group, consistent with existing theory. To improve participants’ cognitive functions, future studies should employ a regular SPB training paradigm.

To investigate the neuromechanism underlying these neuromodulation techniques and further enhance efficiency, numerous studies have tried to combine interventions. For instance, anodal tDCS combined with repetitive peripheral nerve stimulation (rPNS) has been shown to promote motor hand recovery in stroke patients (Sattler et al., 2015). In another study, tDCS combined with tRNS, which is another method of transcranial stimulation, exhibited better facilitation of WM (Murphy et al., 2020). Synchronized intermittent theta burst stimulation (iTBS) and invasive vagus nerve stimulation (VNS) have been shown to be safe, feasible, and potentially effective depression treatments (George et al., 2020). In a recent study, we demonstrated that combined taVNS with tDCS evoked greater activation in a range of cortical and subcortical regions (Sun et al., 2021b), and increased the consistency of WM improvements in healthy participants (Zhao et al., 2022). Since SPB has been shown to have various benefits for physical and mental health and is growing more popular in western society, many researchers have tried to combine it with neuromodulation techniques. For example, Schlatter et al. (2021) found that combined tDCS with HRV biofeedback (breath at 0.1 frequency) significantly reduced participants’ subjective stress. An insightful review pointed out that, considering the similarity of the mechanism and effects of taVNS and SPB, and their possible interactions at the level of the central autonomic network, this kind of hybrid intervention might have potential applications in both clinical and non-clinical areas (Szulczewski, 2022). However, as the various neuromodulation techniques involve complex mechanisms, their combination might induce antagonistic effects (Schabrun et al., 2013). Although a single SPB intervention did not increase participants’ WM performance in the present study, there were no antagonistic effects with taVNS in the taVNS + SPB group, indicating that the underlying mechanism of SPB is not in conflict with taVNS. Thus, although the results of the current study did not reveal a synergistic effect of combined taVNS and SPB interventions, considering the positive influence of SPB on participants’ autonomic system activity (Carter and Carter, 2016; Russo et al., 2017; Niu et al., 2019) and improvements in cognitive performance (Mrazek et al., 2012; Seppala et al., 2014; Quach et al., 2016; Bonomini et al., 2020; Schulz-Heik et al., 2022), this kind of combined intervention (i.e., taVNS + SPB) is still worth investigating in the future. A new combination technique—namely combined respiratory-gated taVNS (RAVNS) with SPB and an intervention-sensitive participant cohort, such as clinical or chronic disease patients—may be two breakthroughs in this area. Furthermore, collecting physiological or neurophysiological indicators, like HRV or electroencephalogram (EEG) data, may be important for quantifying the effects and understanding the underlying mechanism.

Consistent with the effects of breath on the autonomic nervous system, i.e., respiratory gate theory, recent studies have demonstrated that respiratory-gated taVNS, i.e., exhalation-locked taVNS, exerts cardiovagal modulative effects (Garcia et al., 2017; Sclocco et al., 2019) because the nucleus tractus solitarii (NTS), as the primary synaptic target of afference over the vagus nerve, receives an inhibitory influence during inhalation and a facilitatory influence during exhalation (Miyazaki et al., 1998, 1999; Baekey et al., 2010). Several brain imaging studies have confirmed the RAVNS effects, namely that exhalation-locked taVNS can lead to larger regulation of brainstem activity and cardiovagal modulation than inhalation-locked taVNS (Sclocco et al., 2019). Notable, a few of these studies combined RAVNS with a slow respiratory frequency. To date, two studies have examined taVNS with SPB (Frokjaer et al., 2016; Juel et al., 2017), and one combined RAVNS with SPB (Keute et al., 2021); all of these studies reported an increment in HRV. Therefore, one reason for the lack of synergistic effects of taVNS and SPB in this study may be that the independent taVNS intervention without the respiratory phase locked limits interaction and cooperation SPB. To further explore the synergistic effect of taVNS and SPB, both expiration-locked taVNS, i.e., RAVNS, and respiratory frequency should be considered in future studies.

Slow-paced breathing, as one kind of CBT intervention, influences the respiratory system (Bernardi et al., 1998; Nattie and Li, 2012), cardiovascular system (Hsieh et al., 2003; Dick et al., 2014; Ovadia-Blechman et al., 2017), and autonomic nervous system (Lopes and Palmer, 1976; Badra et al., 2001; Eckberg, 2003). Given the complex processes, it may take a long time for effects to accumulate and influence advanced cognitive abilities. This might restrict the instantaneous effects of combined SPB and taVNS intervention on WM performance. Besides, for healthy participants, most of their physiological indicators are normal and stable, which makes it hard for them to reap benefits from the gentle changes in the cardiovascular system and/or autonomic nervous system induced by SPB. In contrast, previous studies suggest that participants suffering from diseases or bad emotion states, such as chronic pain (Jafari et al., 2017; Lehrer et al., 2020; Reneau, 2020), cardiovascular diseases (Russo et al., 2017; Zou et al., 2017; Lehrer et al., 2020), anxiety, depression or stress (Goessl et al., 2017; Zaccaro et al., 2018; Pinter et al., 2019; Lehrer et al., 2020) were sensitive to the effects of SPB. Therefore, to further investigate the synergistic effects of taVNS and SPB, it may be important to recruit different participant cohorts—especially for clinical patients—and evaluate various behavioral measures that include, but are not limited to, ECG, electroencephalogram, emotional tasks, and cognitive tests.

With a growing body of studies supporting the cognitive modulatory effects of taVNS over the last 10 years, many have focused on the underlying mechanisms. However, the detailed processes and mechanisms remain unclear. Regulation of a range of cortical and subcortical regions, including the thalamus, hippocampus, amygdala, and insula (Goehler et al., 2000; Saper, 2002), as well as the ability to activate the locus coeruleus (LC) and cholinergic neurons in the nucleus basalis to further release noradrenaline (NE) and acetylcholine (Hassert et al., 2004; Roosevelt et al., 2006; Nichols et al., 2011), are two plausible mechanisms of taVNS’s cognitive modulatory effects. However, there are likely many undiscovered mechanisms. As a team that has been focusing on taVNS-based WM performance modulation (Sun et al., 2021a,b; Zhao et al., 2023), we have investigated the synergistic effects of taVNS and tDCS and found significant improvement in the modulation effects (Sun et al., 2021a,b). Therefore, the current study explored the synergistic effects of taVNS and SPB on WM performance and we intend to continue investigating their combined effects. We believe that combined taVNS with other neuromodulation techniques or CBT interventions will have great potential for cognitive modulation.

Specifically, we found that the taVNS and taVNS + SPB interventions specifically improved mACC in the spatial 3-back task, but not the spatial 1-back or digit 3-back tasks. This phenomenon has been replicated in more than 300 participants in our previous studies (Sun et al., 2021a; Zhao et al., 2022, 2023), suggesting there may be some special mechanisms. A meta-analysis suggested that the verbal n-back, like the digit n-back task, was associated with enhanced activation in the left ventrolateral prefrontal cortex, whereas the non-verbal location n-back task, like the spatial n-back task, was associated with enhanced activation in a set of regions described as the spatial attention network, which includes the right dorsolateral prefrontal, lateral premotor, and posterior parietal cortex (Owen et al., 2005). Some studies have found that taVNS stimulation can improve selective attention (Sun et al., 2017), which may affect the spatial attention network and contribute to the effect of improved taVNS on spatial WM performance. Participants’ baseline performance may be another influential factor. The effect of modulation of electric field on WM depends on baseline performance (Assecondi et al., 2021). As a result, individuals or tasks with a lower baseline outcome, like the spatial 3-back task, are more likely to show greater improvement. To further understand this phenomenon, more research—especially brain function studies—are required in the future.

Lastly, there are some limitations of the current study. Firstly, although there is a clear physiological rationale for the relationship among taVNS, SPB and the autonomic nerves system, the current study did not test the exact changes of participants autonomic nervous activity, which makes it difficult to draw a conclusion about the changes under the combined interventions of taVNSand SPB and the underlying mechanism of them on WM performance. Thus, the specific mechanisms for taVNS, SPB, and their combined intervention still need research with neurophysiologic or biological indicators to explain in the future. Secondly, to continue exploring a more efficient taVNS technique for WM capacity modulation, we focused on the effects of taVNS, SPB and taVNS + SPB on WM performance. However, both taVNS and SPB influence a wide range of physiological and psychological functions, such as emotion regulation (Strigo and Craig, 2016; Russo et al., 2017; Zaccaro et al., 2018) and pain management (Hassett et al., 2007; Berry et al., 2014; Chakravarthy et al., 2015). Thus, taVNS combined with SPB may have a large potential in these areas. Thirdly, since this study focused on a healthy cohort, it is unknown whether these changes can predict the therapeutic effects of taVNS + SPB-based treatment in different patient groups. Thus, more research is urgently needed to evaluate clinical translation of this technique.

## 5. Conclusion

In summary, informed by previous research, this study concluded that the effects of taVNS on WM accuracy—especially spatial WM with high cognitive loads—were stable and could be replicated in different cohorts. Considering that, taVNS is convenient, easy to administer, and has fewer side-effects, it may be an effective WM modulation technique and thus of important clinical significance. SPB is also a convenient, popular, and effective intervention. Combining these interventions may have a larger effect for cognitive modulation. However, facilitation of SPB and its synergistic effect with taVNS on WM performance was not observed in this. However, we view this study as the beginning of research on this new synergistic technique, not the end. Further research combining RAVNS with SPB using different participant cohorts is required in the future.

## Data availability statement

The raw data supporting the conclusions of this article will be made available by the authors, without undue reservation.

## Ethics statement

This study was approved by the Institutional Research Ethics Committee of Xijing Hospital of the Air Force Medical University. The patients/participants provided their written informed consent to participate in this study. Written informed consent was obtained from the individual(s) for the publication of any potentially identifiable images or data included in this article.

## Author contributions

J-BS, RZ, C-ZT, N-GX, and WQ conceived the study. RZ, Q-QT, HD, X-JY, L-ML, and J-BS designed the study. Z-XY, P-HL, and Y-PC acquired the data. RZ, Q-QT, Z-XY, P-HL, and J-BS analyzed the data. RZ, Q-QT, CC, X-JY, and J-BS drafted the manuscript. All authors revised the manuscript, read, and approved the submitted version.
